# Knowledge, Attitude, and Practice on Pediatric Tuberculosis Management among Healthcare Workers in the Centre Region of Cameroon: A Cross-Sectional Study

**DOI:** 10.1155/2022/4482131

**Published:** 2022-12-15

**Authors:** Thomas Achombwom Vukugah, Derick Akompab Akoku, Micheline Mekemnang Tchoupa, Edward Lambert

**Affiliations:** ^1^Atlantic International University, Honolulu, HI, USA; ^2^Catholic University of Cameroon, Bamenda, Cameroon; ^3^Health Alliance International, Abidjan, Côte d'Ivoire; ^4^Department of Global Health, University of Washington, Seattle, WA, USA; ^5^Ministry of Public Health, Yaoundé, Cameroon

## Abstract

**Background:**

This study was designed to assess the level of knowledge, attitude, and practice (KAP) of healthcare workers (HCWs) on pediatric TB management and its associated factors in the Centre Region of Cameroon.

**Methods:**

A cross-sectional study was conducted between January and March 2022. HCWs, selected through a multistage sampling technique in 21 health facilities, were interviewed using a KAP questionnaire on pediatric TB management. Logistic regression analyses were used to test associations between HCWs' characteristics and knowledge, attitudes, and practice levels at a 0.05 level of significance.

**Results:**

The median age of the participants was 35 years (IQR = 30–42), and the majority (73.2%) were females. About half (50.9%) of the participants (173/340) had good knowledge, 55.6% (189/340) had a good attitude, and 57.1% (194/340) had good practice scores on pediatric TB management. Having a bachelor's degree and above, working in the TB unit, and having received training on pediatric TB in the last five years were significantly associated with good knowledge of pediatric TB management. Similarly, having a bachelor's degree or higher and more than five years of experience providing TB services were significantly associated with a good attitude towards pediatric TB management. Being a general practitioner, nurse, and lab technician was significantly associated with good practice in pediatric TB management.

**Conclusion:**

The level of knowledge, attitude, and practice on pediatric TB management among HCWs was suboptimal, as substantial gaps were identified. The Ministry of Health and other international organizations need to prioritize training, coaching, and mentoring support to help HCWs improve their knowledge, attitude, and practice to detect, diagnose, and treat pediatric TB.

## 1. Introduction

Pediatric tuberculosis (TB) is a major public health challenge and has not been given high priority by the National TB Control Programs (NTCP) because children are believed to be less likely to transmit the disease [1, 2]. TB in children is a direct consequence of adult TB and is a good indicator of current community transmission [3]. From 2018 to 2019, approximately 500,000 children were diagnosed and notified of having TB globally, which represents approximately 11% of the total TB caseload [4]. Infants, young children, and HIV-positive children are more likely to develop TB after being infected with *Mycobacterium tuberculosis* (M.tb) [5, 6] and have a higher risk of severe TB disease and death than adults [6, 7].

In Cameroon, TB is a major cause of mortality among people living with human immunodeficiency virus (PLHIV), and Cameroon is included on the global list of TB high-burden countries by the WHO from 2021 to 2025 [8]. The HIV epidemic has exacerbated TB in the country, as it is the most common opportunistic infection among PLHIV. In the general population, the prevalence of HIV is estimated to be 2.7% [9]. Among the 22,499 cases of all forms of TB detected in 2020, 1,158 (5.2%) were diagnosed among children (<15 years), which suggests that 50% of pediatric TB cases are not diagnosed [10].

Cameroon's 2019 revised TB guidelines provide guidance and direction on the screening and diagnosis of TB in children. These guidelines also include algorithms to help healthcare workers (HCWs) identify symptoms, diagnose pediatric TB, and follow up on children with suspected TB [11]. Based on these revised guidelines, the most effective procedures for reducing prevalence, drug resistance, and poor treatment outcomes for pediatric TB include timely diagnosis, efficient reporting, follow-up, and a new case identification system [12, 13]. Effective performance of pediatric patient-managed care programs is often reliant on the number, distribution, knowledge, skills, levels of motivation, and competence of supporting HCWs [14]. HCWs play an important role in the global fight against TB, although they have a heightened risk of being infected with the disease given their exposure to TB patients [15–17]. These individuals are expected to have adequate knowledge, skills, and competencies to properly detect and manage pediatric TB cases.

Some studies have reported that the limited knowledge of HCWs remains a barrier to the diagnosis, treatment, and prevention of pediatric TB [18–20]. Even though pediatric TB is of public health significance in Cameroon, very limited studies have been conducted to examine the knowledge, attitude, and practice (KAP) of HCWs providing pediatric TB care and treatment. As a result, there are limited data on the knowledge and skill levels of HCWs providing TB management among children. The lack of such data makes it challenging for the Ministry of Health (MOH) and the NTCP to determine which areas to target capacity-strengthening programs for HCWs.

The objectives of this study were to (1) assess the level of knowledge, attitude, and practice of HCWs on pediatric TB management and its associated factors and (2) determine if there is a correlation between knowledge, attitude, and practice among HCWs in the Centre Region of Cameroon. This study will identify capacity-building needs and barriers in program delivery to assist HCWs in providing quality pediatric TB care.

## 2. Methods

### 2.1. Study Setting

This study was conducted in the Centre Region of Cameroon because it is one of the three regions in the country with the highest burden of TB cases. The littoral, centre, and far north regions accounted for 45% of all notified TB cases in Cameroon in 2018 [21]. There are many health facilities of various categories (general hospitals, central hospitals, regional hospitals, district hospitals, district medical centres, integrated health centres, ambulatory health centres, etc.) in the region, especially in Yaoundé, which is the largest city. The Centre Region has 55 TB diagnosis and treatment centres (DTCs) and eight TB treatment centres (TCs). As a result, TB activities are implemented in 63 health facilities across the region.

### 2.2. Study Design and Population

This study was a multicenter cross-sectional study. The study population was frontline HCWs providing TB services across health facilities in the Centre Region of Cameroon. Frontline HCWs who have worked in TB care and management for at least two years, including pulmonologists, pediatricians, general practitioners, nurses, lab technicians, midwives, and allied professionals, and are willing to provide informed consent, were eligible for the study. All HCWs working in the area of TB who were not physically fit at the time of the study and those who refused to provide informed consent were excluded from the study.

### 2.3. Sampling of the Health Facilities

The list of the 63 health facilities known as Diagnostic and Treatment Centres (DTCs) in the region was considered the sampling frame. We applied a modified version of the multistage sampling technique to select 21 health facilities where data were collected. The 1st stage and 2nd stage of the sampling have been described in another published study [22]. Briefly, in the 1st stage, all the DTCs were stratified into public, private, and faith-based categories. At the end of the stratification, there were 38 health facilities in the public category, seven in the private category, and 18 in the faith-based category.

Based on time and available resources, the study team agreed that data should be collected from one-third (*n* = 21) of the 63 DTCs in the region. Consequently, in the 2nd stage, simple random sampling (using the lottery method) was used to select health facilities from each of the three categories based on a probability proportional to size. At the end of the random selection process, a total of 21 health facilities (13 public, two private, and six faith-based) were selected to participate in this study.

In the 3rd stage, a mapping was conducted in each of the 21 health facilities drawn from the 3 strata. The aim was to establish a list of HCWs by category and number in each of these health facilities. This listing was done in collaboration with authorities at the health facilities. From each category, a simple random sampling of HCWs was done based on a probability proportional to the size of the HCW category per health facility. This sampling procedure was used to ensure that the HCWs who participated in the study represented the different categories of HCWs providing TB care at the health facility to a greater extent.

### 2.4. Sample Size Determination

The sample size for the study was calculated based on the formula of sample size calculation for cross-sectional studies, as follows:

(1) $$\begin{array}{c} n=\frac{\left[z_{2}²\;z_{2}\;p\left(1-p\right)\right]}{\left[m_{2}\right]}=\frac{\left[{\left(1.96\right)}_{2}²\;x\;0.5\left(1-0.5\right)\right]}{\left[0.05_{2}\right]}=384HCWs\text{,} \end{array}$$

where *n* is the sample size, *z* = 1.96 is the critical value of the confidence interval for a standard normal distribution (for 95% confidence intervals). *P* = 0.5 is an estimated response rate (as this expected proportion *p* produces the largest sample size (for a given value of m). The value of 50% has been used because at the time of this study, there was no previous study on KAP related to pediatric TB care and management among HCWs in Cameroon, and *m* = 0.05 is the required precision.

Assuming a nonresponse rate of 5% (19 HCWs), the minimum estimated total sample size was 19 + 384 = 403 HCWs.

### 2.5. Development of Data Collection Tool

The study questionnaire was developed in English after reviewing published literature [15, 23], the union course on pediatric TB, and the WHO guidelines for designing TB KAP surveys [24]. After an initial draft of the questionnaire was designed, it was improved using feedback and opinions from a team including clinicians, a social scientist, and an epidemiologist with experience in TB and KAP studies. Thereafter, the questionnaire was translated into French and back-translated into English to ensure coherence and consistency. The French version was necessary because the study setting is predominantly French-speaking. Finally, the questionnaire was pilot-tested among a sample of 25 HCWs providing TB care in three health facilities, which were not selected to participate in the final study. Based on the pilot study, the questionnaire was modified, with some questions reworded to ease understanding. All the questions were adjusted to make them culturally sensitive.

The final questionnaire consisted of 50 questions divided into 4 sections. The first section collected data on respondents' sociodemographic characteristics (e.g., age, sex, level of education, role in the facility, and time spent in the facility); the second section assessed pediatric TB knowledge among respondents; the third section assessed respondents' attitudes towards pediatric TB management; and the fourth section assessed respondents' practices in pediatric TB management. Responses for Sections 2–4 were categorical and/or ordinal. Based on the data from the pilot testing of the questionnaire, the Cronbach's alpha for attitude and practice was 0.69 and 0.87, respectively. The study team ensured that the items shared covariance and measured the same underlying concept.

### 2.6. Data Collection Process

We conducted the study between January and March 2022. Before data collection, three research assistants (data collectors) were hired and trained on the study objectives, recruitment of study participants, obtaining informed consent, and use of the data collection tools. The research team worked with the general supervisor of each health facility to identify the working days of the selected HCWs. This was important as it enabled the data collectors to know the days to visit the health facility and collect data from the eligible HCWs. The data collectors paid a courtesy visit to each health facility to seek permission for data collection. Once granted, data were collected through an interviewer-administered questionnaire with the frontline HCWs working at the TB unit, HIV service, pediatric service, vaccination unit, laboratory service, and outpatient department (OPD) after obtaining written informed consent. The principal investigator supervised the overall activities through continuous supportive supervision. At the end of each day, all questionnaires were reviewed for completeness and consistency. Each data collector transferred the data in the questionnaires to an electronic tablet using ODK software, which was uploaded to a Google server.

### 2.7. Variables of Interest and Measures

Dependent variables: the main dependent variables were knowledge, attitude, and practice in pediatric TB management. We used the median score as the cut-off value for all the questionnaires. Those with a total score below the median were classified as having poor knowledge, attitudes, and practices, while those with a total score equal to or above the median were considered to have good knowledge, attitudes, and practices.

- Knowledge: knowledge of pediatric TB was assessed with 20 questions, and each question had “true,” “false,” and “do not know” response options. For the 13 positively worded questions, all the “true” responses were given a score of “1,” while the “false” and “do not know” responses were given a score of “0.” For the 7 reverse-coded questions, all the “false” responses were given a score of “1,” and the “true” and “do not know” responses were given a score of “0.” Questions not answered were given a score of “0.” The total knowledge score ranged from 0 to 20. The sum of knowledge scores was dichotomized based on the median, which was 14.0. Respondents with a total score equal to or above the median were considered to have good knowledge and were coded as “1,” while those with a total score less than the median were considered to have poor knowledge and were coded as “0.”
- Attitude: attitude towards pediatric TB (Cronbach's alpha = 0.69) was measured using 10 items (6 positively worded and 4 negatively worded) assessing HCWs' attitudes towards pediatric TB transmission, diagnosis, and information regarding pediatric TB. The response options were measured on a 5-point Likert scale with strongly agree (5 points), agree (4 points), neutral (3 points), disagree (2 points), and strongly disagree (1 point). We reversed the scores of the negatively worded statements and then added the total score for attitude. The total score ranged from 10 to 50, and the median value (34.0) of attitude response was considered as the cut-off value to code attitude response to “1,” which indicated having a good attitude regarding pediatric TB if the response sum was greater than or equal to the median. Similarly, the attitude response was coded “0,” indicating having a poor attitude.
- Practice: pediatric TB practice (Cronbach's alpha = 0.87) included 10 items (7 positively worded and 3 negatively worded) regarding the use of face masks and the practice of other precautionary measures. The statements related to practice had the following scores and response choices: 1 = never, 2 = sometimes, and 3 = always. We reversed the scores of the negatively worded statements and then added the total score for practice. The practice statement's total score ranges from 10 to 30, and the median, which was 24.0, was computed to code participants' responses to “1,” indicating having good practice towards pediatric TB if the response was greater or equal to the median, otherwise having poor practice.

The independent variables included age, sex, level of education, professional category, number of years of work experience in the health sector, number of years of work experience in TB care, current working unit, previous training on pediatric TB, and health facility type.

### 2.8. Statistical Analysis

Data were downloaded in Excel format from the Google server, verified for accuracy and consistency, and then imported to IBM's SPSS software version 24.0 (Armonk, NY, USA) for analysis. Continuous variables were measured as means and standard deviations, while categorical variables were expressed as frequencies and proportions. Inferential statistics were applied depending on the nature of the data and variables.

Multivariate logistic regression analyses were performed to determine the strength of the association between the dependent variables (knowledge, attitude, and practice) and the independent variables in a full model. The independent variables were selected for the final model purposefully based on the literature review [18, 19, 25] and the desirability of the authors. In the multivariate model, controlling for potential confounders, there was no evidence of multicollinearity among the independent variables. An adjusted odds ratio (AOR) with 95% confidence levels (CI) was used to quantify the strength of the association. Pearson-rank correlation tests were performed to determine any correlation between the knowledge, attitude, and practice of HCWs regarding pediatric TB management. The statistical significance for all tests was set at *p* < 0.05.

#### 2.8.1. Ethical Considerations

Ethical approval for this study was granted by the Centre Region Ethical Committee for Human Health Research with reference number CE N0 031/CRERSH/2022. Administrative approval was obtained from each of the selected health facilities and the Centre Regional Delegation for Public Health. All participants gave their written informed consent before the interviews, and participation in the KAP study was voluntary. The questionnaires were completely anonymous and did not include any data that could be used to identify the respondents.

## 3. Results

Of the 403 respondents approached, 340 expressed an interest in participating, giving a response rate of 84.4%. The most common reason for refusal was a lack of time to participate in the study, given the busy schedules of HCWs at the health facility.  Table 1 shows the sociodemographic characteristics of study participants. The median age of the participants was 35 years (IQR = 30–42), and the majority were females (73.2%). Most (193, 56.8%) of the respondents had at least a bachelor's degree. The majority were nurses (164, 48.2% and 107, 31.5%) working in the outpatient unit. Also, about half (54.4%) had worked in the health facility for more than five years, and 251 (73.8%) had provided TB care for between 2 and 5 years. Only 140 (41.2%) of the HCWs had received training on pediatric TB in the last five years.

### 3.1. Knowledge of HCWs on Pediatric TB Management

Table 2 shows the knowledge of HCWs on pediatric TB management. The results showed that 173 (50.1%) HCWs had good knowledge, while 167 (49.1%) had poor knowledge of pediatric TB management. Most (282, 82.9%) of the respondents were aware of persistent cough for more than 14 days as a typical symptom of TB in children. Only 140 (40.2%) knew that pulmonary TB is more common in children than extrapulmonary TB. Only half (173, 50.9%) of the participants could identify induced sputum as an appropriate specimen for the diagnosis of TB in children.

### 3.2. The Attitude of HCWs towards Pediatric TB Management

Table 3 shows the attitude of HCWs towards TB management among children. Of the total respondents, 189 (55.6%) had a good attitude towards pediatric TB management, while the rest (151) (44.4%) had a poor attitude towards pediatric TB management. One hundred and twenty-one (35.5%) of the HCWs “strongly disagreed” or “disagreed” to continue to socialize with a child if he/she was diagnosed with TB, and 165 (46.8%) of the HCWs “strongly agreed” or “agreed” that they would recommend a chest X-ray for a presumptive child who is negative for gene Xpert. Detailed attitude results are presented in Table 3.

### 3.3. Practices of HCWs on Pediatric TB Management

Table 4 shows the practices of HCWs towards pediatric TB management. The results showed that 194 (57.1%) HCWs had good practice, while 146 (42.9%) had poor practice in pediatric TB management. Only 91 (26.8%) of HCWs separate coughing children from other children during a consultation, and 173 (50.9%) of HCWs reported that they always wear a mask while consulting children with suspected TB. Only 94 (27.6%) of the HCWs always perform community contact tracing. Detailed practice results are presented in Table 4.

### 3.4. Association between the Demographic Characteristics and Knowledge, Attitude, and Practice in Pediatric TB Management

Table 5 shows adjusted models for the association between the demographic characteristics and knowledge, attitude, and practice in pediatric TB management. In multivariate analysis, HCWs with a bachelor's degree and above were more likely (AOR = 2.61; 95% CI, 1.20–5.66, *p* = 0.015) to have had good knowledge on pediatric TB compared to those with at most a high school certificate. HCWs working in the TB unit were more likely (AOR = 7.26; 95% CI, 2.07–43.60, *p* = 0.010) to have good knowledge on pediatric TB than those working in any other unit. HCWs who had received training on pediatric TB management in the last five years were more likely (AOR = 3.63; 95% CI, 2.15–6.10, *p* < 0.001) to have had good knowledge than those who did not receive any training.

HCWs with a bachelor's degree and above were more likely (AOR = 1.99; 95% CI, 0.98–4.05, *p* = 0.045) to have had a good attitude towards pediatric TB management than those with at most a high school certificate. HCWs with more than five years of experience providing TB services were more likely (AOR = 0.56; 95% CI, 0.34–0.94, *p* = 0.029) to have a had good attitude towards pediatric TB management than those with between 2 and 5 years of experience.

HCWs who are general practitioners (AOR = 6.49; 95% CI, 2.74–15.36, *p* < 0.001), nurses (AOR = 2.71; 95% CI, 1.35–5.39, *p* = 0.005), and lab technicians (AOR = 2.86; 95% CI, 1.29–6.34, *p* = 0.010) were more likely to employ good practice on pediatric TB management compared to those in the other professional category.

### 3.5. Correlation Analysis between Knowledge, Attitude, and Practice

Table 6 shows the correlation analysis between knowledge, attitude, and practice. All three variable scores (knowledge, attitude, and practice) were normally distributed, and therefore, we conducted a Pearson-rank correlation. We found a significant positive but weak correlation between knowledge score and practice score (*r* = 0.199, *p* < 0.001), knowledge score, and attitude score (*r* = 0.174, *p* = 0.001). Moreover, there was also a weak but significant positive correlation between attitude and practice scores (*r* = 0.130, *p* = 0.017).

## 4. Discussion

This study was designed to assess the level of knowledge, attitude, and practice of HCWs on pediatric TB management and its associated factors in the Centre Region of Cameroon. This KAP study found suboptimal knowledge, attitude, and practice on pediatric TB management among HCWs. Overall, only half (50.9%) of the HCWs had good knowledge on pediatric TB management. Our findings are similar to those of previous studies conducted in South Arabia [26] and Uganda [27], which also reported that 52% of HCWs had good knowledge on pediatric TB management. However, the proportion of HCWs with good knowledge found in our study was lower compared to studies conducted in Gabon (79.7%) [15] and Peru (67.3%) [28]. The difference may be due to the variation in study design, methods, and the tools that were used to measure knowledge in these studies [29].

This study found that 82.9% (283/340) of HCWs knew that persistent cough for more than 14 days is a typical symptom of TB in children, similar to findings from a study in Cambodia (86.8%) [19] and higher than a study in Ethiopia (68.5%) [30] but lower than a study in Nigeria (99.5%) [31]. Also, in our study, 90.6% (308/340) of HCWs knew that HIV-infected children have a high risk of exposure to TB infection and disease. This finding is similar to that in Ethiopia (87%) [30] but higher than in a study in Nigeria (77%) [31]. This difference in knowledge might be due to the different target populations selected for the studies. In our study, HCWs were selected from both referral hospitals and health centres, whereas participants in the previous study were selected only from referral hospitals. In addition, our study found that 55.3% (205/340) of HCWs indicated that a negative biological test (e.g., microscopy, gene Xpert, or culture) is an indication of no TB in children. These findings are similar to those of studies conducted in Mozambique [18] and Cote d'Ivoire [27], which found that less than 30% of respondents could identify that Xpert MTB/RIF® was a test used to diagnose TB and less than 50% of HCWs knew that Xpert is a molecular test for TB, respectively.

In this study, training on pediatric TB management in the last five years was significantly associated with good knowledge on pediatric TB management. HCWs who had received training on pediatric TB in the last five years had significantly higher knowledge levels than their colleagues who did not receive such training. Previous studies conducted in Gabon [15] and Mozambique [18] reported similar factors influencing pediatric TB knowledge among HCWs. This finding highlights the importance of providing HCWs with training opportunities (e.g., refresher training, regular in-service training programs by external institutions, etc.) in pediatric TB management. There is scientific evidence that the capacity-building of frontline HCWs contributes to improved childhood TB management [25].

Our study found that overall, 44.4% (151/340) of HCWs had a poor attitude towards pediatric TB management. This was observed in key areas like recommending a chest X-ray for a presumptive child who is negative for Gene Xpert (36.7% disagreed and strongly disagreed); continuing to socialize with a child if he/she was diagnosed with TB (35.5% disagreed and strongly disagreed); bringing close contacts of index cases for TB testing (34.4% disagreed and strongly disagreed); and sharing the same cutlery, plates, and glasses with a family member infected with TB (44.4% disagreed and strongly disagreed). The poor attitudes found in this study might be due to the low proportion of trained and experienced HCWs providing TB services in the respective health facilities. This implies that there are common gaps in attitudes among some HCWs on how TB is transmitted and diagnosed, and similar findings have been previously reported in Cameroon [32] and Gabon [15].

This study found that HCWs with more than five years of experience in pediatric TB management were significantly more likely to have a good attitude towards pediatric TB management than their colleagues with less than five years of experience. Previous studies conducted in Ghana [33], Saudi Arabia [26], and South Africa [34] have demonstrated that the number of years of experience HCWs have in pediatric TB management has shown to positively influence HCWs' attitudes. This could be because, in the years of implementing childhood TB activities, HCWs learn from the job and from colleagues through experience sharing; thus, they are more likely to have a good attitude towards pediatric TB management. This may explain why a majority of the HCWs with less than five years of experience in childhood TB care stated that they would like to learn more about childhood TB.

Our study found that 57.1% (194/340) of HCWs had good practice in pediatric TB management, and similar results have previously been reported in studies conducted in Mozambique [18] and Ethiopia [29]. There were several poor practices identified among HCWs in this study, which is consistent with findings from previous studies [15, 35, 36]. For example, during consultation, 23.5% (80/340) of HCWs did not separate children who were coughing from those who had no cough. Furthermore, 10.3% (35/340) of HCWs are not always wearing a mask when consulting TB-suspected children. These findings are not surprising, as in Cameroon and other settings in sub-Saharan Africa, there is a lack of resources (equipment, space, sufficient medical supplies, etc.), particularly in peripheral health facilities for pediatric TB control and management [15]. HCWs are prone to employing poor practices when these resources are lacking, as it hinders their ability to be efficient and effective at work.

In this study, there was a significant association between the HCWs' profession and pediatric TB practice, which is consistent with a previously conducted study in Mozambique [18]. In this study, general practitioners were more likely to employ good practices in pediatric TB management than nurses and lab technicians. General practitioners have a better level of education given their thorough medical training, they are more likely to have experience in childhood TB as they participate more in seminars and workshops on pediatric TB. Thus, the lessons learned and experience shared on childhood TB among each other makes them more likely to employ good pediatric TB practices.

The findings of this study should be interpreted taking into consideration some limitations. Firstly, this study was cross-sectional, so the associations found do not necessarily infer a causal relationship. Secondly, we relied on self-reported attitudes and practices; hence, there may have been an overestimation or underestimation of good attitudes and practices among HCWs. Lastly, the findings of the study cannot be generalized to the entire frontline HCWs providing TB services in Cameroon. Despite these limitations, the study has gathered information on where gaps exist with regard to the knowledge, attitude, and practices on pediatric TB management among HCWs. These findings are useful for the MOH and international organizations working on pediatric TB in Cameroon. It underscores the need for the MOH and these organizations to allocate substantial resources to strengthen the health system by enhancing pediatric TB training, and strengthening the capacity of HCWs, and ensuring equitable access to medical equipment and supplies. Further studies should be conducted at the national level with a qualitative component so that the practices of HCWs would be observed while providing pediatric TB management. The barriers and facilitators affecting childhood TB diagnosis among HCWs in Cameroon are other areas for research. Additionally, the agreement and disagreement among frontline HCWs providing TB services regarding attitudes present significant areas for further study that may have the potential to highlight particular issues with the NTCP.

## 5. Conclusion

The level of knowledge, attitude, and practice of pediatric TB management was suboptimal, as substantial gaps were identified. A higher level of education, current work in the TB unit, and pediatric TB-related training were significantly associated with good knowledge, whereas experience in providing TB services and a higher level of education were the independent factors associated with a good attitude. Being a general practitioner, nurse, or lab technician was significantly associated with good practice in pediatric TB management. The Ministry of Health and other international organizations need to prioritize training, coaching, and mentoring support from experienced HCWs to help them improve their level of knowledge, attitudes, and practices concerning pediatric TB management.

## Figures and Tables

**Table 1:** Sociodemographic characteristics of the study population.

Characteristics	Frequency (*N*)	Percentage (%)
Age		
21–30	91	26.8
31–40	151	44.4
40+	98	28.8
Sex		
Female	249	73.2
Male	91	26.80
Education level		
High school and below	104	30.6
Diploma^#^	43	12.6
Bachelor's degree and above	193	56.8
Profession		
Pediatrician	10	2.9
General practitioner	58	17.1
Nurse	164	48.2
Lab technician	62	18.2
Others^*∗*^	46	13.5
Current working unit		
Pediatric	31	9.1
Outpatient unit	107	31.5
TB unit	41	12.1
HIV service	66	19.4
Vaccination	32	9.4
Laboratory	51	15
Others^*π*^	12	3.5
Years of experience in a health facility		
0–5 years	155	45.6
6 years+	185	54.4
Years of experience providing TB services		
2–5 years	251	73.8
6 years+	89	26.2
Received TB training in the past 5 years		
Yes	140	41.2
No	200	58.8
Health facility type		
Public	222	65.3
Private	24	7.1
Faith-based	94	27.6

^#^Diploma, two years of post-high school studies; ^*∗*^other professions, including pulmonologists, psychosocial agents, data clerks, and midwives; ^*π*^other working units, including maternity, pharmacy, and surgery.

**Table 2:** Knowledge of HCWs on pediatric TB management.

Pediatric TB knowledge questions	True, *N* (%)	False, *N* (%)	Do not know, *N* (%)
TB epidemiology			
TB is transmitted by a mosquito bite	0 (0)	337 (99.1)	3 (0.9)
Close contact with an index case is not a risk for developing TB in children	32 (9.4)	275 (80.9)	33 (9.7)
TB is no longer a health threat to children because there are medications available	148 (43.5)	174 (51.2)	18 (5.3)
Persistent cough for more than 14 days is a typical symptom of TB in children	282 (82.9)	40 (11.8)	18 (5.3)
TB diagnosis			
Extrapulmonary TB is more common than pulmonary TB in children	119 (35.0)	140 (40.2)	81 (23.8)
Chest X-ray remains an important tool for the diagnosis of pulmonary TB in children	295 (86.8)	28 (8.2)	17 (5.0)
The induced sputum is not an appropriate specimen for the diagnosis of TB in children	103 (30.3)	173 (50.9)	64 (18.8)
A negative biological test (e.g., microscopy, Gene-Xpert, or culture) is an indication of no TB in children	135 (39.7)	152 (44.7)	53 (15.6)
TB treatment			
The recommended therapeutic regimen for childhood TB in Cameroon is 2 (RHZE)/7 (RH)	113 (33.2)	114 (33.5)	113 (33.2)
Treatment outcome is not important data for monitoring and evaluation	36 (10.6)	253 (74.4)	51 (15.0)
During TB treatment in children, the most important adverse reaction is urine with an orange colour	142 (41.8)	91 (26.8)	107 (31.5)
During the treatment of childhood TB, dosages are calculated according to age	177 (52.1)	137 (40.3)	26 (7.6)
TB coinfection			
In case of TB/HIV coinfection, TB treatment and ART must be initiated at the same time	80 (23.5)	227 (66.8)	33 (9.7)
Nutritional support is not necessary for TB/HIV coinfected children	18 (5.3)	303 (89.1)	19 (5.6)
HIV-infected children have a high risk of exposure to TB infection and disease	308 (90.6)	21 (6.2)	11 (3.2)
All HIV-infected children should be regularly screened for symptoms of possible TB	295 (86.8)	26 (7.6)	19 (5.6)
TB prevention			
BCG protects children for life against TB	102 (30.0)	222 (65.3)	16 (4.7)
Non-TB-infected contacts under 5 years of age are eligible for preventive treatment	236 (69.4)	56 (16.5)	48 (14.1)
All contact cases with symptoms of TB must not be referred to the hospital for clinical examination	21 (6.2)	305 (89.7)	14 (4.1)
Infection control measures are not an approach implemented in health facilities to prevent TB in children	41 (12.1)	230 (67.6)	69 (20.3)
Overall knowledge level	Good knowledge 173 (50.9)		
	Poor knowledge 167 (49.1)		

**Table 3:** Attitude of HCWs towards pediatric TB management.

Attitude items	Strongly agree	Agree	Neutral	Disagree	Strongly disagree
Diagnostic tools in this health facility are adequate for the diagnosis of childhood TB	87 (25.6)	74 (22.8)	75 (22.1)	68 (20.0)	36 (10.6)
Laboratory services in this health facility are adequate for the diagnosis of childhood TB	50 (14.7)	101 (29.7)	56 (16.5)	66 (19.7)	66 (19.4)
I would continue to socialize with a child if he/she was diagnosed with TB	75 (22.1)	73 (21.5)	71 (20.9)	78 (22.9)	43 (12.6)
I can share the same cutlery, plates, and glasses with family a member if he/she was infected with TB	73 (21.5)	75 (22.1)	41 (12.1)	68 (20.0)	83 (24.4)
I would be willing to learn more about childhood TB	133 (39.1)	60 (17.6)	33 (9.7)	28 (8.2)	86 (25.3)
I am willing to get my child screened for TB regularly	95 (27.9)	104 (30.6)	65 (19.1)	40 (11.8)	36 (10.6)
Always insufficient dispersible pediatric drugs to treat childhood TB in the health facility	82 (24.1)	89 (26.2)	94 (27.6)	42 (12.4)	33 (9.7)
I would ask smear-positive pulmonary TB patients to bring their close contact to the health facility for TB screening	127 (37.4)	69 (20.3)	27 (7.9)	31 (9.1)	86 (25.3)
I would recommend a chest X-ray for a presumptive child who is negative for gene Xpert	86 (26.2)	79 (20.6)	56 (16.5)	47 (13.8)	78 (22.9)
I would refer suspected TB children for TB diagnostic workup	116 (34.1)	80 (23.5)	35 (10.3)	26 (7.7)	83 (24.4)
Overall attitude	Good attitude	189 (55.6)			
	Poor attitude	151 (44.4)			

**Table 4:** Practices of HCWs on pediatric TB management.

Practice item	Always, *N* (%)	Sometimes, *N* (%)	Never, *N* (%)
I suspect TB in a child who has been coughing for more than 14 days	146 (42.9)	139 (40.9)	55 (16.2)
I separate coughing children from other children during a consultation	91 (26.8)	169 (49.8)	80 (23.5)
I wear a mask when consulting TB's suspected children	173 (50.9)	132 (38.8)	35 (10.3)
I educate TB-suspected children on how to cough	170 (50.0)	129 (39.9)	41 (12.1)
I open windows when a TB-suspected child is in the room	168 (49.4)	121 (35.6)	51 (15.0)
I give priority to children coughing in the waiting area	119 (35.0)	160 (47.1)	61 (17.9)
Contact tracing is performed at a community level	94 (27.6)	182 (53.5)	64 (18.8)
If a child is diagnosed with TB, I give the parent/guardian relevant information about the disease	206 (60.6)	112 (32.9)	22 (6.5)
I systematically screen for TB symptoms in children during a consultation	139 (40.9)	165 (48.5)	36 (10.6)
I follow the national TB treatment guidelines to treat TB in children	163 (47.9)	129 (37.9)	48 (14.1)
Overall practice	Good practice	194 (57.1)	
	Poor practice	146 (42.9)	

**Table 5:** Association between the demographic characteristics and knowledge, attitude, and practice in pediatric TB management.

ALL variables	Knowledge		Attitude		Practice	
	AOR (95% CI)	*P* value	AOR (95% CI)	*P* value	AOR (95% CI)	*P* value
Age						
21–30	1.0 (reference)		1.0 (reference)		1.0 (reference)	
31–40	1.42 (0.69–2.91)	0.343	0.61 (0.32–1.14)	0.126	0.94 (0.53–1.64)	0.816
40+	1.29 (0.50–3.31)	0.595	0.60 (0.27–1.34)	0.219	1.52 (0.81–2.92)	0.187
Sex						
Female	0.80 (0.42–1.50)	0.494	0.78 (0.46–1.33)	0.376	1.41 (0.81–2.44)	0.217
Male	1.0 (reference)		1.0 (reference)		1.0 (reference)	
Education level						
High school and below	1.0 (reference)		1.0 (reference)		1.0 (reference)	
Diploma^#^	0.53 (0.22–1.25)	0.151	1.07 (0.51–2.27)	0.847	0.92 (0.44–1.92)	0.840
Bachelor's degree and above	2.61 (1.20–5.66)	**0.015**	1.99 (0.98–4.05)	**0.045**	1.39 (0.64–2.99)	0.401
Profession						
Pediatrician	2.85 (0.37–21.87)	0.312	2.96 (0.47–18.48)	0.245	3.10 (0.76–12.66)	0.115
General practitioner	2.62 (0.92–7.41)	0.069	1.82 (0.68–4.89)	0.232	6.49 (2.74–15.36)	**<0.001**
Nurse	0.98 (0.43–2.77)	0.973	1.17 (0.54–2.50)	0.680	2.71 (1.35–5.39)	**0.005**
Lab technician	0.74 (0.16–3.61)	0.747	1.56 (0.36–6.71)	0.549	2.86 (1.29–6.34)	**0.010**
Others^*∗*^	1.0 (reference)		1.0 (reference)		1.0 (reference)	
Current working unit						
Pediatric	2.88 (0.64–12.97)	0.158	1.57 (0.38–6.39)	0.529	3.21 (0.64–16.06)	0.155
Outpatient unit	2.02 (0.61–8.55)	0.290	1.02 (0.29–3.61)	0.965	2.31 (0.59–9.00)	0.227
TB unit	7.26 (2.07–43.60)	**0.010**	0.95 (0.24–9.37)	0.942	3.23 (0.74–14.00)	0.116
HIV service	2.80 (0.89–14.42)	0.141	1.77 (0.47–6.54)	0.392	4.00 (0.97–16.39)	0.054
Vaccination	0.43 (0.12–3.18)	0.311	2.67 (0.63–11.34)	0.181	1.57 (0.34–7.14)	0.559
Laboratory	1.52 (0.38–6.08)	0.553	1.55 (0.40–5.93)	0.517	5.91 (0.92–37.91)	0.061
Others^*π*^	1.0 (reference)		1.0 (reference)		1.0 (reference)	
Years of experience in a health facility						
0–5 years	1.0 (reference)		1.0 (reference)		1.0 (reference)	
6 years+	0.64 (0.37–1.10)	0.138	1.14 (0.68–1.92)	0.607	0.67 (0.37–1.19)	0.175
Years of experience providing TB services						
2–5 years	1.0 (reference)		1.0 (reference)		1.0 (Reference)	
6 years+	1.62 (0.85–3.06)	0.108	0.56 (0.34–0.94)	**0.029**	1.10 (0.61–1.99)	0.730
Received TB training in last 5 years						
Yes	3.63 (2.15–6.10)	**<0.001**	1.52 (0.95–2.45)	0.078	1.18 (0.71–1.96)	0.504
No	1.0 (reference)		1.0 (reference)		1.0 (reference)	
Health facility type						
Public	0.92 (0.48–1.72)	0.788	0.42 (0.17–1.05)	0.065	0.76 (0.43–1.33)	0.343
Private	1.01 (0.34–2.96)	0.982	.99 (0.01–0.1)	0.991	1.11 (0.39–3.16)	0.839
Faith-based	1.0 (reference)		1.0 (reference)		1.0 (reference)	

^#^Diploma, two years of post-high school studies; ^*∗*^other professions, including pulmonologists, psychosocial agents, data clerks, and midwives; ^*π*^other working units, including maternity, pharmacy, and surgery.

**Table 6:** Correlation analysis of knowledge, attitude, and practice.

Variable	Correlation coefficient	*P* value
Knowledge-practice	0.199^*∗*^	<0.001
Knowledge-attitude	0.174^*∗*^	0.001
Attitude-practice	0.130^*∗*^	0.017

^*∗*^Correlation is significant at 0.05 level (2-tailed).

## Data Availability

The data used to support the findings of this study are available from the corresponding author upon request.

## Supplementary Materials

Supplementary Materials Study questionnaire: The final questionnaire consisted of 50 questions divided into 4 sections (see supplementary appendix). The first section collected data on respondents' sociodemographic characteristics; the second section assessed pediatric TB knowledge among respondents; the third section assessed respondents' attitudes towards pediatric TB management; and the fourth section assessed respondents' practices in pediatric TB management.
