# Supplementary material for: Knowledge, Attitude, and Practice on Pediatric Tuberculosis Management among Healthcare Workers in the Centre Region of Cameroon: A Cross-Sectional Study
Source: J Trop Med. 2022 Dec 15;2022:4482131. doi: 10.1155/2022/4482131 (PMC9780008; doi:10.1155/2022/4482131)
Supplement: Supplementary Materials — Study questionnaire: The final questionnaire consisted of 50 questions divided into 4 sections (see supplementary appendix). The first section collected data on respondents' sociodemographic characteristics; the second section assessed pediatric TB knowledge among respondents; the third section assessed respondents' attitudes towards pediatric TB management; and the fourth section assessed respondents' practices in pediatric TB management. [file 4482131.f1.docx]

**QUESTIONNAIRE KAP STUDY**

**Knowledge, Attitude, and Practice on pediatric TB management among frontline Health care Personnel in Centre Region of Cameroon**

| **Instructions:** We appreciate if you give us some background information about yourself |
| --- |
| **A1. Age in years as of last birthday**___________________ |
| **A2. Sex**  ❒_1_ Female ❒_2_ Male |
| **A3. How long have you been working as a health care professional?**  ❒_1_ 0-5 years  ❒_2_ 6 years+ |
| **A4 How long have you been providing TB services in this health facility?**  ❒_1_ 2-5 years  ❒_2_ 6 years+ |
| **A5. Which of the following best describes your profession?**  ❒_1_ Pediatrician ❒_2_ General Practitioner ❒_3_ Nurse  ❒_4_ Lab technician ❒_5_ Others (Please specify) _____________ |
| **A6. What is your highest level of Academic Qualification?**  ❒_1_ High school and below ❒_2_ Diploma ❒_3_ Bachelor Degree and above |
| **A7. In which service do you work at this health facility?**  ❒_1_ Pediatric service ❒_2_ Outpatient Department ❒_3_ TB Unit ❒_4_ HIV Service ❒_5_ Vaccination  ❒_6_ Laboratory ___________ ❒_6_ Others (Please specify) ___________ |
| **A8. Have you ever received training on pediatric TB in the past 5 years?**  ❒_1_ Yes (Skip to Question A9) ❒_2_ No |
| **A9 What was the training duration?**  ❒_1_ < 3 days ❒_2_ 3-5 days ❒_3_ 6 days and above |

**SECTION 2: KNOWLEDGE ON CHILDHOOD TB**

| ***Instructions: This section relates to your knowledge on pediatric TB Management. Please indicate if you believe the statements are true or false. (Please tick ☑ ONLY ONE BOX per statement).*** | | | |
| --- | --- | --- | --- |
| **QUESTIONS ON KNOWLEDGE** | True | False | Don’t know |
| 1. **EPIDEMIOLOGY** |  |  |  |
| **K1.** TB is transmitted by a mosquito bite | **❒_3_** | **❒_2_** | **❒_1_** |
| **K2.** Close contact with an index case is not a risk for developing TB in children | **❒_3_** | **❒_2_** | **❒_1_** |
| **K3.** TB is no longer a health threat to children because there are medications available | **❒_3_** | **❒_2_** | **❒_1_** |
| **K4.** Persistent cough for more than 14 days is a typical symptom for TB in children | **❒_3_** | **❒_2_** | **❒_1_** |
| 1. **DIAGNOSIS** |  |  |  |
| **K5.** Extra- pulmonary TB is more common than pulmonary TB in children | **❒_3_** | **❒_2_** | **❒_1_** |
| **K6**. Chest X-ray remains an important tool for the diagnosis of pulmonary TB in children | **❒_3_** | **❒_2_** | **❒_1_** |
| **K7.** The induced sputum is not an appropriate specimen for the diagnosis of TB in children | **❒_3_** | **❒_2_** | **❒_1_** |
| **K8.** A negative biological test (e.g., microscopy, gene xpert or culture) is an indication of no TB in children | **❒_3_** | **❒_2_** | **❒_1_** |
| 1. **TREATMENT** |  |  |  |
| **K9** The recommended therapeutic regimen for childhood TB in Cameroon is  2 (RHZE) / 7 (RH) | **❒_3_** | **❒_2_** | **❒_1_** |
| **K10** Treatment outcome is not are important data for monitoring and evaluation | **❒_3_** | **❒_2_** | **❒_1_** |
| **K11** During TB treatment in children, the most important adverse reaction is urine with an orange colour | **❒_3_** | **❒_2_** | **❒_1_** |
| **K12** During the treatment of childhood TB, dosages are calculated according to age | **❒_3_** | **❒_2_** | **❒_1_** |
| 1. **CO-INFECTION** |  |  |  |
| **K13**. In case of TB/HIV co-infection, TB treatment and ART must be initiated at the same time | **❒_3_** | **❒_2_** | **❒_1_** |
| **K14.** Nutritional support is not necessary for TB/HIV co-infected children. | **❒_3_** | **❒_2_** | **❒_1_** |
| **K15**. HIV infected children have a high risk of exposure to TB infection and disease | **❒_3_** | **❒_2_** | **❒_1_** |
| **K16.** All HIV-infected children should be regularly screened for symptoms of possible TB | **❒_3_** | **❒_2_** | **❒_1_** |
| 1. **PREVENTION** |  |  |  |
| **K17.** BCG protects children for life against TB | **❒_3_** | **❒_2_** | **❒_1_** |
| **K18.** Non-TB infected contacts under 5 years of age are eligible for Preventive Treatment | **❒_3_** | **❒_2_** | **❒_1_** |
| **K19.** All contact cases with symptoms of TB must not be referred to hospital for clinical examination | **❒_3_** | **❒_2_** | **❒_1_** |
| **K20.** Infection control measures is not an approach implemented in health facilities to prevent TB in children | **❒_3_** | **❒_2_** | **❒_1_** |

**SECTION 3: ATTITUDE**

| ***Instructions: This section relates to your attitudes*** *on Pediatric TB Management. How strongly do you agree or disagree with each of the following statements* ***(Please tick*** *☑*  ***ONLY ONE BOX*** *per statement)*  ***Key: SA=Strongly Agree; A=Agree; N=Neutral; D=Disagree and SD=Strongly Disagree*** | | | | | | |
| --- | --- | --- | --- | --- | --- | --- |
| **CODE** | **ATTITUDES ON CHILDHOOD TB** | **SD**  **(1)** | **D**  **(2)** | **N**  **(3)** | **A**  **(4)** | **SA**  **(5)** |
| A1 | Diagnostic tools in this health facilities are inadequate for diagnosis of childhood TB | **❒_1_** | **❒_2_** | **❒_3_** | **❒_4_** | **❒_5_** |
| A2 | Laboratory services in this health facility are adequate for diagnosis of childhood TB | **❒_1_** | **❒_2_** | **❒_3_** | **❒_4_** | **❒_5_** |
| A3 | I would continue to socialize with a child, if he/she was diagnosed with TB | **❒_1_** | **❒_2_** | **❒_3_** | **❒_4_** | **❒_5_** |
| A4 | I cannot share same cutlery, plates and glasses with a family member, if he/she was infected with TB | **❒_1_** | **❒_2_** | **❒_3_** | **❒_4_** | **❒_5_** |
| A5 | I would be willing to learn more about childhood TB | **❒_1_** | **❒_2_** | **❒_3_** | **❒_4_** | **❒_5_** |
| A6 | I am not willing to get my child screened for TB regularly | **❒_1_** | **❒_2_** | **❒_3_** | **❒_4_** | **❒_5_** |
| A7 | Always insufficient dispersible pediatric drugs to treat childhood TB in the health facility | **❒_1_** | **❒_2_** | **❒_3_** | **❒_4_** | **❒_5_** |
| A8 | I Would ask smear positive pulmonary TB patient to bring their close contact to health facility for TB screening | **❒_1_** | **❒_2_** | **❒_3_** | **❒_4_** | **❒_5_** |
| A9 | I would recommend chest x ray for a presumptive child who is negative for GeneXpert? | **❒_1_** | **❒_2_** | **❒_3_** | **❒_4_** | **❒_5_** |
| A10 | I would refer suspected TB children for TB diagnostic work up | **❒_1_** | **❒_2_** | **❒_3_** | **❒_4_** | **❒_5_** |

**SECTION 4: PRACTICE**

| ***Instructions: This section relates to your practice regarding childhood TB.*** ***Please indicate how often you do the following. (Please kindly tick  ONLY ONE box per statement*** | | | |
| --- | --- | --- | --- |
| **CHILDHOOD TB PRACTICES** | **Always** | **Sometimes** | **Never** |
| **P1.** I suspect TB for a child who has been coughing for more than 14 days | **❒_3_** | **❒_2_** | **❒_1_** |
| **P2**. I separate coughing children from other children during consultation | **❒_3_** | **❒_2_** | **❒_1_** |
| **P3.** I wear a mask when consulting TB suspected children. | **❒_3_** | **❒_2_** | **❒_1_** |
| **P4**. I educate TB suspected children on how to cough | **❒_3_** | **❒_2_** | **❒_1_** |
| **P5.** I open windows when a TB suspected children is in the room | **❒_3_** | **❒_2_** | **❒_1_** |
| **P6.** I don’t give priority to children coughing in the waiting area | **❒_3_** | **❒_2_** | **❒_1_** |
| **P7.** Contact tracing is not performed at community level | **❒_3_** | **❒_2_** | **❒_1_** |
| **P8.** If a child is diagnosed with TB, I give the parent/guardian relevant information about the disease | **❒_3_** | **❒_2_** | **❒_1_** |
| **P9**. I systematically screen for TB symptoms in children during consultation | **❒_3_** | **❒_2_** | **❒_1_** |
| **P10.** I don’t follow the National TB treatment guidelines to treat TB in children | **❒_3_** | **❒_2_** | **❒_1_** |

Thank you for taking the time to participate in this survey. Your responses will be very helpful in answering the research questions which may lead to improvement of pediatric TB control in Cameroon.
